# A rare case of Creutzfeldt‐Jakob disease reported from Nepal

**DOI:** 10.1002/ccr3.4804

**Published:** 2021-09-13

**Authors:** Durga Neupane, Prashant Kumar Gupta, Sushil Sharma Subedi, Dilip Gupta, Sunit Chhetri

**Affiliations:** ^1^ B. P. Koirala Institute of Health Sciences Dharan Nepal; ^2^ Department of Radiology National Academy of Medical Sciences Bir Hospital Kathmandu Nepal; ^3^ B. P. Koirala Institute of Health Sciences Department of Surgery Dharan Nepal

**Keywords:** Creutzfeldt‐Jakob disease, dementia, myoclonus, sporadic

## Abstract

Creutzfeldt‐Jakob disease, though rare, should be considered in the clinical picture of rapidly progressive dementia and absence of verbal response as evident in our case despite the absence of typical radiological picture.

## Graphical abstract

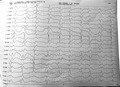

## 1 INTRODUCTION

Creutzfeldt‐Jakob disease (CJD) is a rare disorder of the central nervous system. It is rapidly progressive and always fatal. Even in absence of typical imaging pattern, good clinical judgement along with supporting investigations can aid to the diagnosis of CJD in a setting where resources are limited.

We report a rare case of probable Creutzfeldt‐Jakob disease (CJD) in a 65‐year‐old man, probably the second case in Nepal, who initially presented with progressively increasing low mood with catatonia along with rapidly progressive dementia and features of upper motor neuron lesions. The first case of CJD from Nepal being reported by Kharel et al^1^in 2019. Magnetic resonance imaging of brain revealed confluent areas of T2 and fluid‐attenuated inversion recovery (FLAIR) high signal intensity in bilateral fronto‐parietal deep white matter. The electroencephalogram showed bilaterally synchronous periodic pattern of bi‐ or triphasic sharp waves of 1 Hz. The patient expired at 1.5 months of diagnosis.

Creutzfeldt‐Jakob disease is a progressive, fatal neurodegenerative disease and is caused by misfolded, transmissible proteinaceous infections particles, or prions. Although the concentration of CJD prions varies throughout the body of an infected individual, it is highest in the brain and the posterior eye (retina and optic nerve),^2^, ^3^ resulting in neurological symptoms, including rapidly progressing dementia, cerebellar and extrapyramidal signs, and myoclonus and visual symptoms. The life expectancy of most people clinically diagnosed with CJD is 1 year from the onset of symptom.^2^ Among the three major groups of human prion disease: sporadic, genetic, and acquired; sporadic CJD (sCJD) is most common, accounting for about 85% of CJD cases.^4^ With the occurrence generally in late middle age at a mean age of 67 years, they have a short survival post‐diagnosis of about 4 months. However, at least six different clinic‐pathological CJD subtypes with variable presentations are known.^5^, ^6^ Even with the evidence of a genetic predisposition to sCJD,^7^ the precise cause of the disorder is unknown. The global incidence of CJD is typically reported to be around 1–2 cases per million per year.^8^

We present a case report that includes the clinical and radiological features of the probably second case of CJD in Nepal, and also illustrates the complexity of diagnosing this disease in a resource‐limited setting.

## 2 CASE REPORT

A 65‐year‐old man nondiabetic normotensive reformed smoker and ex‐alcohol consumer with known case of ulcerative colitis and benign prostatic hyperplasia (under medications for 2 years) visited our center with complaints of decreased verbal output, decreased psychomotor activity and low mood for the last 1.5 months. The symptoms started in the form of memory loss (forgetting address, phone numbers, to turn on the lights in the evening, to locate headlight of the bike) with progression to the right‐sided hand stiffness and ipsilateral leg weakness 3 months back. He then visited nearby medical center and was prescribed with medications (Escitalopram and Quetiapine), which did not improve his symptoms and his condition deteriorated further. Later on, he also developed a sudden difficulty of his speech with reduced speech output and inability to speak normally on the thirty‐fifth day of the onset of his symptoms. Ten days prior to presentation to our hospital, he had no verbal output, no oral intake, inability to walk without support, and loss of control in urine and stool passage.

He had a history of disturbed sleep pattern for the last 1 month. There were features of low mood and non‐compliant behavior at the time of presentation. He did not have a history of loss of consciousness or head trauma. There was no history of fever, swelling of limbs, headache, tremor, visual loss, seizures, weight loss, and exposure to toxic substances. There was no history of drug abuse. He was a non‐vegetarian and had no history of psychiatric disorders. There was no similar history in the family.

On examination, the vital signs were stable. The Glasgow Coma Scale was E4V1M5, and pupils were bilaterally equal and reactive. The fundus examination was normal. He had patchy whitish lesion over dorsum of tongue and erythematous plaque over perineum. He had no signs of lymphadenopathy, meningism. Muscle tone was increased in all four extremities. Bilateral biceps, triceps, and knee reflexes were 3+. Planter reflexes were upgoing bilaterally. He also showed wide‐based gait. Respiratory, cardiovascular, and abdominal examinations were normal.

A complete blood count, hemoglobin, erythrocyte sedimentation rate, coagulation profile, liver, and renal function tests, C‐reactive protein, serum electrolytes (Na^+^, K^+^, Ca^+2^, and Mg+^2^), serum glucose, and urinalysis were within normal range. Chest X‐ray and Mantoux tests were also normal. Antinuclear antibody, complements C3, C4 were found to be negative. Cerebrospinal fluid (CSF) parameters showed no abnormality and adenosine deaminase in CSF was also within a normal range. CSF culture was sterile. Opening pressure during lumbar puncture was not elevated.

**FIGURE 1:**
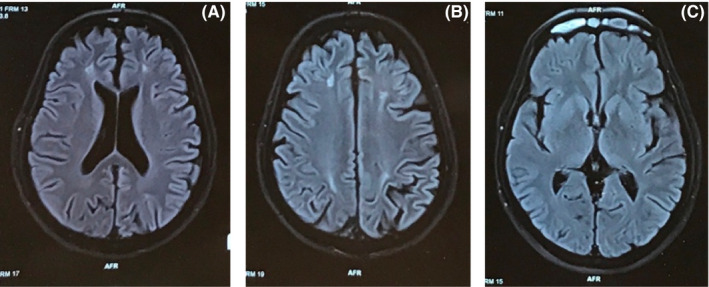
(A) Axial fluid‐attenuated inversion recovery (FLAIR) MRI brain image shows ill‐defined hyperintense areas in deep white matter of bilateral frontal lobes. (B) Axial fluid‐attenuated inversion recovery (FLAIR) MRI brain image shows ill‐defined hyperintense areas in deep white matter of bilateral frontal and parietal lobes. (C) Axial fluid‐attenuated inversion recovery (FLAIR) MRI brain image shows normal bilateral basal ganglia, thalamus, and cortical gray matter

**FIGURE 2:**
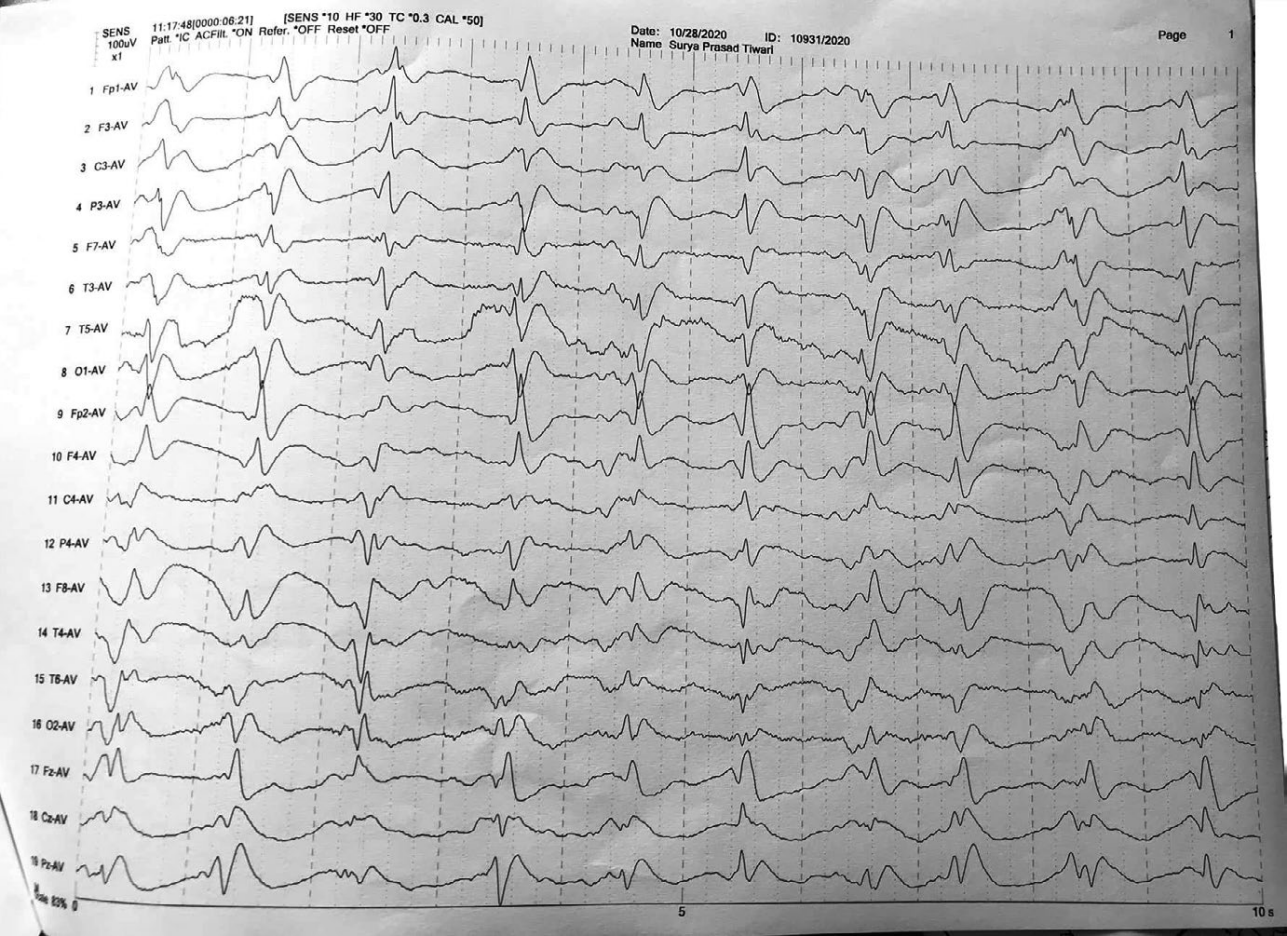
EEG pattern with generalized slow waves, and bilaterally synchronous periodic patterns of bi‐ or triphasic waves of 1 Hz

T2‐weighted magnetic resonance imaging (MRI) and FLAIR showed areas of high signal intensities in bilateral fronto‐parietal lobe deep white matter (Figure 1). The electroencephalogram showed bilaterally synchronous periodic pattern of bi‐ or triphasic sharp waves at 1 Hz (Figure 2). Investigation of CSF 14–3–3 was not available in our center.

The diagnosis of probable CJD was made. Supportive care with anti‐epileptics and antidepressants was provided. The patient was regularly followed up and his condition progressively worsened and died within 1.5 months of diagnosis.

## 3 DISCUSSION

Creutzfeldt‐Jakob disease has a wide phenotypic spectrum in regard to the age of onset, presenting features, rate of progression, and appearance of other clinical manifestations. Although usually presented with various and unspecific symptoms and signs, most patients develop dementia, ataxia and/or visual signs, myoclonus, and muscle tone abnormalities during the course of the disease.^9^

Diagnostic criteria have been updated to identify this rare disorder. Centres for Disease Control and Prevention (CDC)'s diagnostic criteria for CJD, 2018^10^ for probable CJD necessitate a history of progressive neurological syndrome with a positive real‐time quaking‐induced conversion (RT‐QuIC) test. In alternative to the above criteria, rapidly progressive cognitive impairment with at least two out of the following four clinical features: myoclonus, visual or cerebellar symptoms, pyramidal or extrapyramidal symptoms, or akinetic mutism; and at least a positive finding on EEG (generalized periodic complexes), MRI or CSF (positive 14–3–3 protein) and without alternative diagnosis in routine investigations also indicate the diagnosis of probable CJD. Most of the criteria as mentioned by CDC was fulfilled in our case with no alternative diagnosis indicating the probable diagnosis of CJD in our case.

Magnetic resonance imaging is the imaging modality of choice in CJD with sensitivity and specificity of 96% and 93%, respectively.^11^ The most common MRI finding in CJD comprises of cortical and basal ganglia involvement with hyperintensity at DWI in the insula and cingulate (limbic lobe), in addition to involvement of the superior frontal gyri and the cortical areas near the midline.^12^ Although sCJD is generally considered to affect primarily gray matter, and MR imaging abnormalities generally have not been observed in white matter during the early to middle stages, there is histopathologic evidence that white matter damage occurs, characterized by diffuse reactive astrocytic gliosis, activated microglia, and rare PrP^Sc^deposition and vacuolation.^13^ No MRI findings suggestive of prions disease were present in our case possibly because of the early presentation. Therefore, MRI findings were not correlated with the clinical findings in our patient.

Routine CSF analysis for cell count, protein, and oligo‐clonal bands (usually positive in steroid‐responsive encephalitis associated with autoimmune thyroiditis (SREAT), autoimmune encephalitis, viral encephalitis, and multiple sclerosis) is normal in CJD.^14^, ^15^, ^16^ This was consistent in our case as well.

The typical EEG appearances in sCJD comprise of periodic, bi‐ phasic, or triphasic sharp‐wave complexes of 1–2 Hz. These features present late in the disease process.^17^, ^18^ In addition to the sensitivity (64%–66%) and specificity (74%–91%) in diagnosing sCJD, Periodic sharp‐wave complexes (PSWCs) can also differentiate probable sCJD from other prion diseases.^19^, ^20^ However, PSWCs were also recorded in Alzheimer's disease, vascular dementia, and dementia with Lewy body.^20^, ^21^ Therefore, neuroimaging is helpful in distinguishing prion‐related from nonprion‐related rapidly progressing dementia^22^ and is the best predictor for sCJD.^23^ Our case was supported by PSWCs in EEG.

A new diagnostic modality known as RT‐QuIC has remarkably high sensitivity and specificity in recent studies (sensitivity of 85.7% and specificity of 100%) in sCJD.^24^Cerebrospinal fluid biomarkers like 14–3–3, S100 beta, neuron‐specific enolase, and total tau which are considered to be the markers of neuronal injury,^25^ have a controversial role in the diagnosis of sCJD. They have varying sensitivity and specificity and are not prion‐specific proteins.

Making the utilization of investigations available in our center along with the consideration of the clinical presentations, we ruled out many other infectious, iatrogenic, metastatic/neoplasm related, vascular/ischemic, toxic/metabolic, autoimmune, systemic/seizures/sarcoid, and demyelinating causes of rapidly progressive dementia.^26^

Our patient had a rapidly progressive cognitive impairment, myoclonus, and akinetic mutism meeting the diagnostic criteria as mentioned above. Typical EEG pattern with non‐typical MRI pattern supported by the clinical features confirmed the clinical diagnosis of CJD. Thus, our case meets the criteria of probable CJD. Due to the unavailability of CSF 14–3–3 and genetic mutations testing in our setting, they were not performed.

## 4 CONCLUSION

Histopathological examination by the biopsy or autopsy can only lead to the definitive diagnosis of CJD. In a resource‐limited setting, although without typical imaging pattern, clinical and other investigational work up can also be helpful for the diagnosis of such a rare disease. Current study about CJD comprises only the tip of the iceberg. Extensive research is required to reveal all its aspects.

## CONFLICTS OF INTEREST

The authors declare no conflicts of interest.

## AUTHOR CONTRIBUTIONS

DN, PKG, SSS, and DG wrote the initial draft of the manuscript. DN, PKG, and SC edited the draft and reshaped it into this manuscript. Final version of manuscript was approved by all authors and agree to be responsible for all aspects of the work.

## ETHICAL APPROVAL

Consent from patient's son was enough.

## CONSENT

The patient's son provided written informed consent for the publication of this case report and the accompanying images.

## Data Availability

Data openly available in a public repository that issues datasets with DOIs.
